# Three Distinct Isoforms of ATP Synthase Subunit c Are Expressed in *T. brucei* and Assembled into the Mitochondrial ATP Synthase Complex

**DOI:** 10.1371/journal.pone.0054039

**Published:** 2013-01-10

**Authors:** Paul E. Gulde, Linda Christen, Silvia V. Brown, Noreen Williams

**Affiliations:** Department of Microbiology and Immunology and Witebsky Center for Microbial Pathogenesis and Immunology, University at Buffalo, The State University of New York, Buffalo, New York, United States of America; Univ. Georgia, United States of America

## Abstract

One striking feature of the biology of trypanosomes is the changes in mitochondrial structure and function that occur as these parasites transition from one life cycle stage to another. Our laboratory has been interested in the role the mitochondrial ATP synthase plays in mitochondrial changes through the life cycle. Analysis of the recently completed *T. brucei* genome suggested that there may be multiple putative genes encoding ATP synthase subunit c. While homologous in their 3′ ends, these genes differ in their 5′ ends and, if expressed, would result in three distinct proteins. Our analysis showed that all three of the possible transcripts were detected in both procyclic and bloodstream stages, although the c-3 transcript was less abundant than that for c-1 or c-2. The three isoforms of subunit c are produced in both the bloodstream and procyclic stages and their mature protein products possess distinct N-terminal regions of the protein as found within mitochondria. All three isoforms are also incorporated into the assembled ATP synthase complex from procyclic cells. Although multiple subunit c genes have been found in other organisms, they produce identical polypeptides and the finding of significant differences in the mature proteins is unique to *T. brucei*.

## Introduction

Stage coordinated biochemical changes occur throughout the life cycle of *Trypanosoma brucei* allowing the parasite to maximize carbon source usage depending on the environment [1], [2]. This occurs together with vast changes in mitochondrial morphology and protein expression. Procyclic forms found in the midgut of the insect vector maintain a fully functional mitochondrion with well-developed cristae and bioenergetic complexes. The mitochondria within the long slender, early bloodstream cells lack highly developed cristae and a number of the major components of the electron transport chain and TCA cycle. Later, short stumpy bloodstream stages upregulate the expression of several of these complexes. However, the functions of oxidative phosphorylation are not initiated until passage into the insect vector and subsequent development of the procyclic form [2]. Despite these changes in the mitochondria, the ATP synthase is present in all life cycle stages of *T. brucei* [3]. Functional assays and protein analysis have shown that the ATP synthase is moderately developmentally regulated throughout the life cycle of the parasite [4] with the highest levels present in the procyclic stage.

In most eukaryotic cells, the ATP synthase couples the proton gradient generated by the electron transport chain to the synthesis of ATP. The ATP synthase can also work in reverse at high ATP levels and low membrane potential to produce a membrane potential for functions such as calcium transport, mitochondrial import and apoptosis [5]–[8]. Bloodstream *T. brucei* have been shown to maintain a membrane potential despite the lack of electron transport components such as the cytochromes [5], [7], [8]. Studies, including those from this laboratory, have shown that maintenance of the membrane potential in bloodstream *T. brucei* cells is dependent on the presence of a functional ATP synthase [5], [7]–[10].

The ATP synthase complex consists of two major components, the soluble, catalytic F_1_ moiety and the membrane bound, proton pore F_0_ moiety. The *Escherichia coli* F_1_ moiety consists of five subunits in a stoichiometry of α_3_ β_3_ γ_1_ δ_1_ε_1_, and the F_0_ consists of the three subunits in a stoichiometry of a_1_b_2_c_10–14_ [11,12]. While the F_1_ structure is conserved_,_ the number of F_0_ subunits varies among organisms and in mammalian systems there are at least 7 additional subunits beyond the core bacterial components (defg(A6L)F_6_ and OSCP) [11], [13]. Most of the subunits of the eukaryotic ATP synthases are encoded in the nucleus with the exception of subunits a (aka 6), 8, and in some cases, subunit c (aka 9) [11]. The F_1_ moiety in *T. brucei* is also comprised of the five core subunits, while its F_0_ moiety is believed to be similar in complexity to those found in other eukaryotic organisms [14], [15]. OSCP and c were the only non-F_1_ subunits identified from the genome but TAP analysis has suggested a total of 22 subunits in the complex (of which 14 are unique to *T. brucei*) [14].

The core of the ring structure of the F_0_ moiety is formed from multiple copies of the highly hydrophobic subunit c (also known as the DCCD-binding protein). The significance of the c subunit rests in the relationship of the rotation of the ring of c subunits within the membrane, driven by the proton movement across the membrane and down the electrochemical gradient in most cases. The ring contacts the central stalk of the ATP synthase (subunits γδε) whose γ subunit in turn rotates within the α_3_β_3_ headpiece. The change in conformation of the 3 sequential αβ pairs induced by contact with γ allows catalysis to occur [11], [16]. The c ring also contacts subunit a and subunit b, which is part of the stator a critical structure for connecting the stationary parts of the complex. Therefore, both the structure of subunit c and its interactions with other subunits in the complex are critical for ATP synthase function.

The c subunit is comprised of two transmembrane helical segments connected by a short loop to form a hairpin [13], [17], [18]. The most conserved feature of the protein is the c subunit signature sequence, also known as the DCCD binding domain, which contains an essential glutamate in eukaryotes (aspartate in *E. coli*) that binds the inhibitor DCCD (dicyclocarbodiimide). The number of copies of subunit c present in the F_0_ ring has been shown to differ between organisms. In yeast, there are 10 c subunits per ring [19], while 8 are present in mammalian ring structures [13]. The number of c subunits in bacterial systems ranges from 10 to 15 depending on the organism [20]. Previous work suggested that the number of c subunits in *E. coli* changes in response to a change in the carbon source from succinate to glucose, resulting in an increase in the copy number of subunit c in the F_0_ ring [21], although this is observation has not been confirmed [20]. Among mammalian ATP synthases, there are several organisms in which expression of subunit c occurs from multiple gene copies dependent on environmental conditions. However, to date all of the subunit c genes appear to produce the same mature protein [22]–[24].

In this study, we focus on the ATP synthase subunit c of *T. brucei*. We demonstrate that three distinct isoforms of subunit c are expressed and incorporated into the assembled ATP synthase complex. This represents a unique case for ATP synthase complexes and is discussed in view of changes in trypanosome bioenergetics.

## Materials and Methods

### Cell Growth and Antibody Preparation

*T. brucei* strain 427 was used for these experiments (kind gift from Dr. George Cross, Rockefeller University). Bloodstream cells were grown in HMI-1 medium with 10% fetal bovine serum at 37°C [25]. Procyclic cells were grown in Cunningham’s medium with 10% fetal bovine serum at 27°C [26].

Anti-peptide antibodies were prepared against the region of dissimilarity of the putative *T. brucei* ATP synthase subunit c isoforms c-1, c-2, and c-3 (as translated from AAC48310 (Tb11.02.2950), XP_822414 (Tb927.10.1570), and XP_845771 (Tb927.7.1470), respectively, Bethyl Laboratories). The resulting anti-peptide antibodies were assessed for cross-reactivity against the other peptides using dot blot analysis. Using peptides to which the antibodies were raised, 10 µl of a 1 mg/ml peptide preparation was blotted onto nitrocellulose. The nitrocellulose was blocked with a 10% milk in TBST solution. Each antibody was used to challenge each peptide using the Western breeze anti rabbit kit (Life Technologies). No cross reactivity above background was observed at 1∶1000 dilution of the antibodies for the c-1 and c-2 antibodies. However, a small degree of cross reactivity was seen with the c-3 antibody against c-2 peptide at dilutions below 1∶2500 (Figure S1). Antibodies against the control protein β-tubulin were obtained from Chemicon International and antibodies against TbRGG2 were a kind gift from Dr. Laurie Read ([27], University at Buffalo). Antibodies against whole TbF_1_, TbF_0_, and Tb subunit c (previously named S9) were previously described [28], [29].

### Quantitative RT- PCR

Quantitative analysis of the c-1, c-2, and c-3 transcript levels in both the procyclic and bloodstream cell lines was performed using qPCR. Total RNA was isolated from 1×10^8^ cells per ml TRIZOL reagent (Life Technologies). The remaining RNA extraction procedure followed manufacturer directions. The RNA was dissolved in 25 µl DEPC water and its integrity and quantity were assessed by spectrophotometry at 260 and 280 nm as well as analysis by electrophoresis on a 2% TBE agarose gel.

Ten micrograms of RNA were DNAse I treated in a 50 µl reaction following Ambion’s Turbo DNA-free kit directions. Again, RNA quality and quantity were assessed by spectrophotometry and gel electrophoresis. Four micrograms RNA were converted to cDNA using random hexamers (Applied Biosystems) and Superscript III First Strand Synthesis Supermix (Life Technologies) per manufacturer’s recommendations. After cDNA synthesis, the products were incubated with RNAse H (Life Technologies) for 20 minutes at 37°C.

Quantitative RT-PCR standard curves and efficiencies were generated for both the procyclic and bloodstream cDNA using 0.3 µM final concentration gene specific primers (in Table S1) and IQ SYBR Green SuperMix (Bio-Rad). No template and minus reverse transcriptase controls were included for each primer set and cDNA. General amplification conditions using the Bio-Rad MyiQ2 Real-Time detection system were 50°C for 2 min and 90°C for 10 min followed by 40 cycles of 95°C for 15 sec and 60°C for 1 min. Post-amplification melting curves verified single products. Gel electrophoresis confirmed the amplicons’ correct size and DNA sequencing yielded the appropriate isoforms for the specific genes. Results were calculated using iQ5 software (Bio-Rad). The data were normalized to the levels of RNA encoding for actin and Telomerase Reverse Transcriptase (TERT) [30] and the relative differences were calculated using the 2^-ΔΔCt^ (Livak) method [31]. Quantitative results were derived from a minimum of three independent experiments.

#### Western Blot Analysis of Subunit C Isoforms

Procyclic and bloodstream cell cultures were harvested and mitochondria were prepared as previously described [15]. Mitochondrial extracts (100 µg) were solubilized with dodecyl-β-D-maltoside and separated by sodium dodecyl sulfate-polyacrylamide gel electrophoresis. Western blot analysis was then performed using the antibodies directed against the peptides from putative *T. brucei* ATP synthase subunit c isoforms c-1, c-2, and c-3 (as described above) using dilutions of 1∶2000, 1∶1000, and 1∶2500, respectively. Membranes were also probed with a monoclonal anti-β-tubulin as a loading control (Chemicon International). A minimum of 3 biological replicates were performed.

#### Immune Capture Experiments

100 µl Dynabeads (Life Technologies) were cross-linked to anti-c-1, anti-c-2, or anti-c-3 peptide antibodies according to manufacturer’s instructions. Beads alone were used as a negative control for non-specific binding to the Dynabeads (data not shown). A 500 µg aliquot of protein from procyclic and bloodstream mitochondrial extracts was used for each immune capture (IC) sample and was incubated with the beads at 4°C overnight with rotation. Supernatants were collected from experimental reaction mixtures, and beads were washed five times with phosphate-buffered saline (PBS). Beads were resuspended in SDS-polyacrylamide gel electrophoresis buffer and boiled for 5 min. Samples were subjected to SDS-PAGE at 100 V for one hour and then were transferred to nitrocellulose membrane for western blot analysis. Immune capture (IC) reactions using anti-c-1 antibody were analyzed for the presence of c isoform 2 using anti-c-2 antibody and for c isoform 3 using anti-c-3 antibody. Similarly IC reactions using anti-c-2 or anti-c-3 antibody were analyzed for each of the other isoforms using their respective anti-peptide antibodies. ICs were performed with a minimum of three different extracts.

#### High Resolution Clear Native Native Gel Electrophoresis

High resolution clear electrophoresis (hrCNE) was conducted as described by Wittig et al [32]. Mitochondria were prepared as previously described [15], treated for 45 min at RT with deoxyribonuclease (500 U/mg protein, Life Technologies), washed and resuspended in buffer consisting of 50 mM BisTris and 750 mM aminocaproic acid. They were then solubilized using 12.5 µl of 20% dodecyl-β-D-maltoside per 1 mg of protein and incubated at 4°C for 1 hour. Membranous debris was removed by centrifugation at 100,000×g for 15 minutes. Samples (100 µg protein) were mixed with hrCNE loading buffer, applied to 4% to 16% native gels (Life Technologies) and were electrophoresed for 3 hours at 150 V limited to 15 mA. The hrCNE cathode buffer (50 mM tricine, 15 mM Bis-Tris, 0.02% dodecyl-β-D-maltoside, and 0.05% sodium deoxycholate) and anode buffer (50 mM Bis-Tris) were used. The hrCNE experiments were repeated a minimum of four times with different extracts.

#### Western blot analysis of ATP synthase complex

For western blot analysis of the samples separated by hrCNE, gels were treated with 300 mM Tris, 100 mM acetic acid and 1% SDS, pH 8.6, for 20 minutes followed by washing in semi-dry transfer buffer 150 mM Tris and 50 mM acetic acid, pH 8.6. Proteins were then transferred onto nitrocellulose membranes at 15V for 1 hour and western blot analysis was performed using antibodies against the c-1, c-2, and c-3 peptides as well as the control mitochondrial protein TbRGG2 [27], [32]. Antibodies against whole TbF1, TbF0, and Tb subunit c were also used to confirm the identity of the ATP synthase complexes (data not shown).

#### In gel ATPase activity assays

For identification of ATP synthase complexes following hrCNE, gels were incubated overnight in hydrolysis activity assay buffer ([32], 34 mM Tris-HCl, 270 mM glycine, 14 mM MgSO_4_, 4 mM ATP and 0.2% Pb(NO_3_)_2_. The gels were photographed following development of the precipitate.

## Results

### Three Potential Subunit C Genes are Present in the *Trypanosoma brucei* Genome

**Figure 1:**
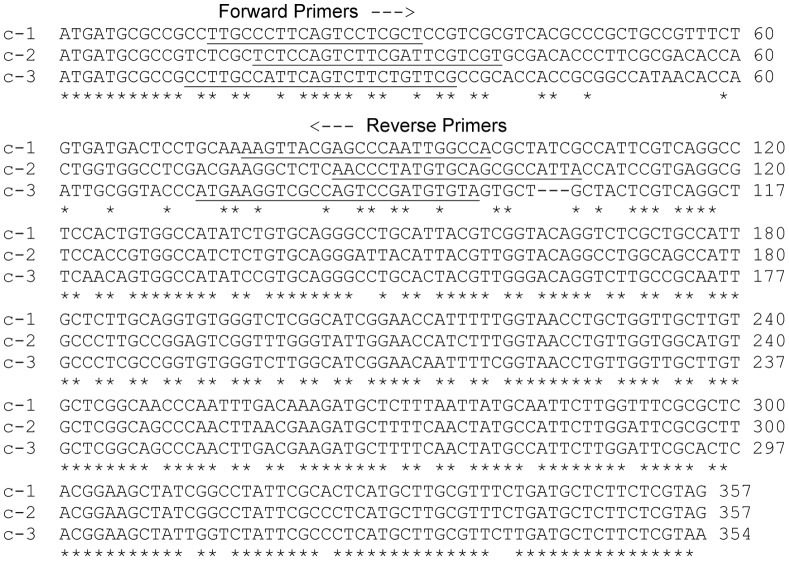
Three potential subunit c genes are present in *T. brucei*. Analysis of the *T. brucei* genome revealed three genes for the ATP synthase subunit c. The genes for the three isoforms contain a region of dissimilarity between nucleotide 45 and 108. Primers designed to target this region are underlined.

Previous work from this laboratory identified a single gene for the ATP synthase subunit c (designated c-1 in Figure 1, accession number AAC48310 (Tb11.02.2950), named S9 in previous publications [29]). This subunit c gene was identified from a transcript amplified using primers for the conserved DCCD-binding domain and the spliced leader sequences. Expression of the encoded protein was confirmed using antibodies raised to both the recombinantly expressed protein and the ATP synthase complex. More recently, the *T. brucei* genome revealed two additional open reading frames that potentially encode subunit c isoforms (accession numbers XP_822414 (Tb927.10.1570), and XP_845771 (Tb927.7.1470), [33], designated c-2 and c-3 in Figure 1). As previously noted [14], the three putative genes show strong identity in the very 5′ region and in the central region to the 3′ end. However, there is a region of significant divergence between nts 45 and 108 (Figure 1).

### *Trypanosoma brucei* Produces Three Distinct Subunit C Transcripts in both Procyclic and Bloodstream Forms of *T.brucei*

**Figure 2:**
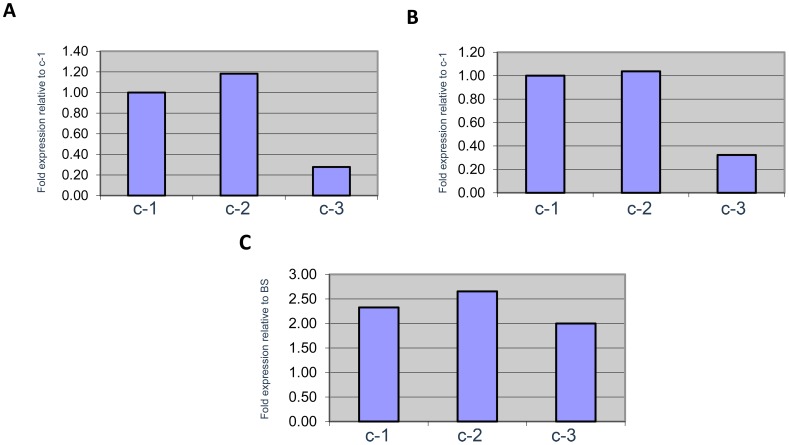
*T. brucei* produces three distinct subunit c transcripts. qRT-PCR analysis of 2 ng procyclic (PC) or bloodstream (BS) cDNA using c-1, c-2, or c-3 gene specific primers (Table S1). A. Expression of c-2 and c-3 relative to c-1 in PC cells. B. Expression of c-2 and c-3 relative to c-1 in BS cells. C. Expression of c-1, c-2, and c-3 in PC cells relative to BS cells.

In order to determine whether the three genes were transcribed, primers were designed to amplify the region of dissimilarity from mRNAs from both procyclic and bloodstream form cells using qRT-PCR (Figure 1, sequences underlined). Using quantitative RT-PCR, mRNA levels of the c-1, c-2, and c-3 genes were analyzed for both procyclic (PC) and bloodstream (BS) life cycle stages of *T. brucei*. In Figure 2, we show that all three potential transcripts were amplified from mRNA prepared from both procyclic and bloodstream cells. Data shown is based on three independent experiments in each case. As seen in Figure 2, the level of c-2 expression relative to c-1 expression is 1.18 in PC (Panel A) and 1.04 in BS (Panel B), slightly higher in both stages but similar to c-1. However, c-1 and c-2 relative gene expression of are 3 to 4 fold greater than that of c-3 at 0.28 (PC, Panel A) and 0.32 (BS, Panel B), respectively. When comparing the mRNA levels of the *atpc* genes between the PC and BS cells, all three c isoforms (Figure 2, Panel C) are expressed at 2-2.5 fold greater levels in PC cells than in BS cells.

### Three Isoforms of Subunit C are Expressed

**Figure 3:**
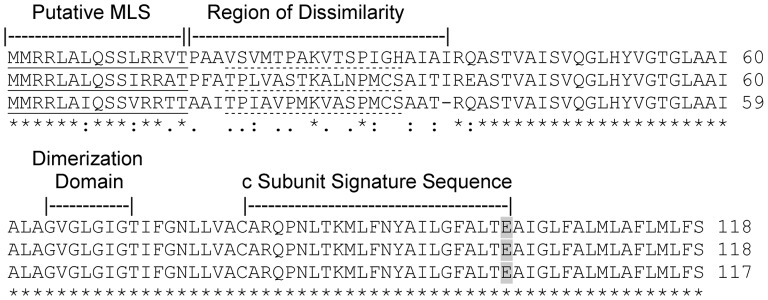
The predicted amino acid sequences for the three putative subunit c proteins are distinct. The putative mitochondrial localization signal (underline), region of dissimilarity, dimerization domain, signature sequence, and essential glutamate (greyed) are indicated. The peptide antibodies employed in the western blot analysis were directed against the dotted underlined sequences in c-1, 2, and 3.

As shown in Figure 3, the three putative subunit c isoforms are identical from aa 39 to the end of the protein sequence. Previous work from this laboratory suggested a 15 aa predicted mitochondrial targeting signal for subunit c-1 based on sequence analysis of trypanosome mitochondrial proteins with known cleaved pre-sequences and the similarity to the iron-sulfur protein targeting sequence (as indicated in Figure 3, [34]–[36]). These predicted mitochondrial targeting sequences for the three c subunits differ by three amino acids (MMRRLALQSSLRRVT and MMRRLALQSSIRRAT, MMRRLAIQSSVRRTT for isoforms c-1, c-2, and c-3, respectively). An alternative cleavage site following aa 40 is predicted by several mitochondrial targeting sequence programs and would generate a longer cleaved presequence, however this is not based on a trypanosome cleavage pattern. Based on the sequences of the pre-cleaved predicted proteins, the three isoforms would have nearly the same molecular weights. The putative isoform c-1 has a predicted molecular weight of 12302 and a pI of 11.38. Putative isoform c-2 has a predicted molecular weight of 12390 and a predicted pI of 10.43. The putative isoform c-3 has a predicted molecular weight of 12189 and a pI of 10.92.

The major region of difference between the three isoforms resides in the sequence from amino acids 15–36 which directly follows the first predicted targeting signal. If the prediction for the cleavage site for mitochondrial import is correct (following the T), the resulting processed proteins would differ in their mature N-terminal sequences. This region of dissimilarity is predicted to occur in an unstructured segment of the proteins which would project into the intermembrane space (MEMSAT3 prediction results, http://bioinf.cs.ucl.ac.uk/memstat/). If the alternative cleavage site is used the resulting proteins would be identical.

We exploited this difference between the three sequences to determine whether the transcripts identified in Figure 2 resulted in three distinct isoforms of subunit c being expressed. Anti-peptide antibodies were prepared to target the region of dissimilarity for each of the putative isoforms (Figure 3, region marked with dotted underlines). The antibodies raised against the c-1 and c-2 peptides do not recognize the other c peptides. The antibody raised against the c-3 peptide showed a low degree of cross reactivity against c-2 peptide when used at lower dilutions (Figure S1) and therefore this antibody was used only at higher dilutions and all results are interpreted conservatively.

**Figure 4:**
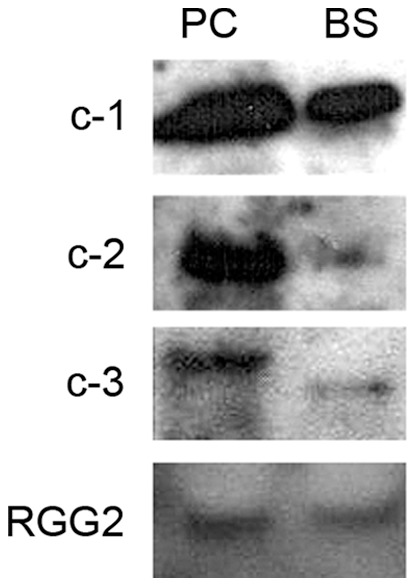
Three isoforms of subunit c are expressed in procyclic and bloodstream form cells. Using anti-peptide antibodies directed to the region of dissimilarity shown in Figure 3, the three isoforms of subunit c were detected by western blot analysis. 100 µg of mitochondrial extract from both procyclic and bloodstream cells were used for detection of c-1, c-2, c-3, and the control mitochondrial protein Tb RGG2.

Western blot analysis of procyclic and bloodstream mitochondrial extracts was performed using each antibody. As shown in Figure 4, all three antibodies, anti-c-1, anti-c-2, and anti-c-3 antibodies, showed strong reactivity with both bloodstream and procyclic extracts. It is clear from these results that c-1, c-2, and c-3 are expressed. Although some low level of cross reactivity is seen with the c-3 antibody, the western blot analysis used antibody at a dilution where little cross reactivity is seen. Therefore, it can be concluded that c-3 is also expressed at the protein level. These results demonstrate that *T. brucei* produces more than one c protein. Furthermore, the results show that the mature forms of the three isoforms still possess the regions of dissimilarity and are therefore most likely cleaved at the first predicted cleavage site (after aa 15), but certainly before the second predicted site (after aa 40).

### The Three Isoforms of Subunit C Interact with each other in Mitochondrial Extracts

**Figure 5:**
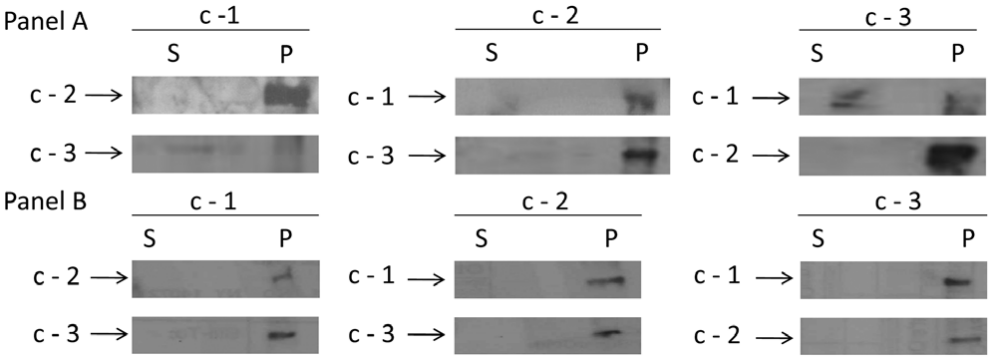
The three isoforms associate with each other in procyclic and bloodstream form *T.* brucei. Immunecapture experiments were performed using protein extracts of procyclic (Panel A) and bloodstream (Panel B) mitochondria with antibodies to each subunit form. Western blot analysis of the immune captured proteins was performed using antibodies against each of the c isoforms.

Mitochondrial extracts from bloodstream and procyclic forms were used in immune capture experiments to determine if the isoforms interact with one another and might be present together in the ATP synthase complex. In procyclic cell mitochondrial extracts, interactions between each of the pairs of isoforms were demonstrated (Figure 5, Panel A), although the interaction between c-1 and c-3 was more difficult to detect than the others (Panel A, left bottom and right top). Similarly, immune capture from bloodstream mitochondrial extracts (Figure 5, Panel B) demonstrated the ability of each isoform to bind with the other two isoforms, although overall the bloodstream form signal was less strong than that of the procyclic form immune captures. This may be due to the overall decreased level of expression of the ATP synthase in bloodstream cells. Attempts to increase the amount of protein employed in western blot analysis does not result in a clearer signal likely due to the hydrophobic nature of the complex.

### All Three Isoforms of Subunit C are Incorporated into the ATP Synthase Complex

**Figure 6:**
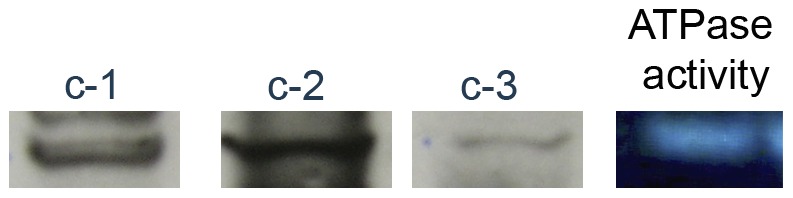
All three isoforms of subunit c are incorporated into the ATP synthase. 100 µg of mitochondrial protein extract from procyclic cells was applied to hrCNE. Lane 4 shows the in gel activity assay. For Lanes 1-3 the proteins were transferred to nitrocellulose and probed for the three subunit c isoforms.

Mitochondrial extracts were subjected to hrCNE to determine whether each of these expressed subunit c isoforms is assembled into the ATP synthase complex. Procyclic cells were used as the source of mitochondria due to the amount of material required. Following hrCNE, samples were transferred to the nitrocellulose membrane and analyzed using the anti-c-1, anti-c-2, and anti-c-3 antibodies. The band representing complete ATP synthase complex was identified using an in gel activity assay (Figure 6, Lane 4) as well as antibodies to whole F_1_, whole F_0,_ and anti-subunit c (data not shown). Western blot analysis using anti-peptide antibodies to each of the isoforms confirms that all three isoforms are present in ATP synthase complexes (Figure 6, Lanes 1–3). These data indicate that each isoform is able to be integrated into the ATP synthase complex, however we were not able to determine whether the isoforms are present in the same complexes or in different complexes within a population.

## Discussion

The *T. brucei* mitochondrial ATP synthase is present in all stages of the parasite life cycle although the levels was 3–5 fold greater in procyclic than bloodstream forms. In the procyclic stage, the mitochondrion is fully active and capable of ATP production by oxidative phosphorylation. In contrast, the replicating long slender bloodstream forms have mitochondria containing minimal cristae, reduced expression of ATP synthase and they lack TCA cycle and electron transport complexes required for oxidative phosphorylation [37]. Differentiation into the non-proliferative stumpy forms begins to re-establish a complex mitochondrion in order for the parasite to survive during the subsequent insect vector stage. The continual presence of ATP synthase despite these vast changes suggests an important and unusual role for this enzyme complex. In fact, previous work has demonstrated that the ATP synthase acts as an ATP hydrolase in the early bloodstream form to produce the mitochondrial membrane potential [5], [7]–[10]. This unusual function might suggest unique structural features within the ATP synthase particularly in regions involved in coupling of proton translocation to catalysis.

This laboratory previously identified a single gene for the ATP synthase subunit c, the core subunit of the proton conducting pore of the complex [29]. The completion of the *T. brucei* genome sequencing revealed two additional putative genes which might allow the expression of three different c proteins (Figures 1 and 3). Most of the central features of the ATP synthase subunit c are identical among the three putative isoforms, however one region of significant difference was identified.

First, the ATP synthase subunit c signature sequence (ExPASY Proteomics server) which contains the essential glutamate required for proton translocation (aspartate in *E.coli*) is identical in all three isoforms. The consensus signature sequence [GSTA]-R-[NQ]-P-x(5)-{A}-x-{F}-x(2)-[LIVMFYW](2)-x(3)-[LIVMFYW]-x-[DE] is A-R-Q-P-N-L-T-K-M-L-F-N-Y-A-I-L-G-F-A-L-T-E in *T. brucei*. The underlined E (glutamate, which is substituted by aspartate in *E. coli*) is also the residue that binds the inhibitor dicyclohexylcarbodiimide (DCCD), which gives this subunit its alternate name, the DCCD-binding protein. This glutamate in the c subunit acts as the proton acceptor and donor at the interface of F_0_ subunit a and provides the ion binding site at each of the c-c interfaces. The subunit c must interact with subunit a then break the interaction to now interact with next c subunit. Subunit a is thought to form two aqueous half channels that allow access to the essential glutamate [38] which is poised within the membrane. Changes in structure of subunit c seem to be limited to the ion binding site [18].

A second critical domain is the polar loop region of c and subunits γδε which form the central stalk. The stalk acts as the internal connection from the c ring to the α_3_β_3_, the catalytic core of the ATP synthase, and so this region of subunit c is part of the connection between the proton conducting ring and the catalytic portion of the complex. This region in both bovine and yeast subunit c is ARNPS [13], [38] and in *T. brucei* is relatively conserved as ARQPN and once again is identical among the isoforms.

A third conserved domain is generally present in the first α helix of subunit c (N-terminal helix which resides on the inner ring) and is a conserved GxGxGxGxG that looks like a dimerization domain for transmembrane α helices [39]. In *T. brucei*, the domain contains only 4 Gs and is a GxGxGxG that is identical in all three of the subunit c isoforms (aa 64 -70). This domain is responsible for the rotor ring stoichiometry and is located in the three dimensional structure opposite the ion binding site [18]. It is responsible for the subunit c-subunit c interaction and the glycines allow the close packing of the subunits. Previous work using site directed mutagenesis of this domain created changes in the packing of the c subunits to increase the copies of the c subunit within the ring structure. This work also demonstrated the functional significance of the size of the ring structure, showing that the c_12_ ring is functional at a lower ion motive force than the c_11_ ring [39].

When we identified the first subunit c gene [29], we postulated that cleavage of a mitochondrial targeting sequence would occur between aa 15 and 16 resulting in a protein of 103 aa. This prediction was based on analysis of mitochondrial membrane proteins from *T. brucei* [34], [36]. We have also examined the putative mitochondrial targeting signal with a number of prediction programs. Most suggested a cleavage site at aa 40. However the presence of the region of dissimilarity suggests this is not the case. If the latter cleavage site at 40 were used, the resulting protein would be 78 aa in length and would not be identified with our anti-peptide antibodies.

Next, we determined that transcripts for *atpc-1, atpc-2,* and *atpc-3* were expressed at detectable levels in both procyclic and bloodstream forms of the parasite (Figure 2). Transcript resulting from *atpc-2* was slightly more abundant than that for *atpc-1* in both life cycle stages, with levels 18 and 4% higher in procyclic and bloodstream forms, respectively. Transcript from *atpc*-3 is 72% less than that of *atpc-1* in PC and 68% less in BS. All three transcripts were higher in procyclic than in bloodstream cells as expected from previous results [29], although the difference is not as great as found in TREU667 bloodstream forms from mice (2-2.5 fold vs. 10-14 fold). We do not know if the difference is due to passage in mice or the strain used.

All three of these transcripts were shown to be translated by western blot analysis using anti-peptide antibodies targeting the region of dissimilarity (Figure 4). The reactivity with these antibodies also demonstrated that the region of dissimilarity between the three isoforms is preserved in the mature proteins within the mitochondria. Among mammalian ATP synthases, there are other examples of multiple copies of subunit c genes which produce functional proteins. In rat and bovine systems, where there are three distinct genes for subunit c, three well conserved isoforms are expressed which are identical in sequence. Differences among the genes reside in the regions encoding cleavable mitochondrial targeting sequences and untranslated regions [22]–[24].The N-terminal mitochondrial targeting sequence is variable in both length and sequence. Recently, the cleaved targeting peptides were shown to play an important role in both structure and function of the respiratory chain [40]. Our results do not show where the cleavage site resides, but it is clearly prior to the 40 aa site and the resulting proteins are longer than the norm for mammalian subunit c. The bovine subunit c has a 61 aa signal peptide and the mature protein is 75 aa in length [13]. The genes, which produce proteins with different import sequences, are expressed in a tissue specific manner [41]. In *S. cerevisiae* the subunit c is mitochondrially encoded and is represented by a single gene which produces a polypeptide of 76 aa [38]. We will try to clarify the N terminal sequence of the three *T. brucei* subunit c isoforms, but this has proven challenging due the hydrophobic nature of these proteins.

Since all three isoforms were expressed at the protein level, we next determined whether they might interact with each other or were present in the extract as homomeric complexes. Immune capture experiments showed that each isoform was in contact with the other two isoforms, although the interaction between isoform c-1 and c-3 was less prevalent. These results indicated that the isoforms might be present in complexes containing multiple isoforms rather than in complexes with only a single isoform.

High resolution clear native gel electrophoresis was conducted to determine whether each subunit c isoform is present in the ATP synthase complex. Samples subjected to hrCNE and analyzed using antibodies to the three isoforms demonstrated that all isoforms are able to integrate into ATP synthase complexes (Figure 6). However, due to the similar size of each subunit, it is not possible to determine the number of each isoform within the complex by mass spectrometry [42]. Future experiments will utilize electron microscopy to address this question. Work from the Stuart laboratory has attempted to characterize the components of the complete ATP synthase in *T. brucei* [14] using several different approaches. Only a single method provided any data regarding subunit c identifying a peptide representing the isoform here designated as isoform c-2 [14]. The reason for this is unclear but may be due to the limitations of mass spectrometry with small hydrophobic molecules.

The significance of the three isoforms of subunit c present in the ATP synthase is not yet known. We have shown that the relative abundance of the three transcripts and the three isoforms differ between the two life cycle stages. We considered whether the ATP synthase complex might have a different number of subunit c polypeptides present in the ring structure of F_0_ depending on the life cycle stage. However, since the GxGxGxG domain has been shown to determine the packing of subunit c within the ring and all of these sequences are identical within the three isoforms, it seems probable that the ring size would remain the same.

In the case of *T. brucei*, the ATP synthase shifts in function from utilizing the membrane potential for the formation of ATP to hydrolysis of ATP to generate a membrane potential. It may be that the change in the relative ratios of the three isoforms of subunit c is important to the function of the ATP synthase in different life cycle stages. It is possible that the difference in the sequence in the region directly after the cleavage of the targeting sequence, which is predicted to reside in the intermembrane space, might be important for interaction with some of the trypanosome specific components of the ATP synthase [14]. This novel interaction could be important for regulation of the activity of the ATP synthase or in the coupling of proton translocation to the catalytic activity in the F_1_ headpiece.

Results from RNAi experiments ([43] and the TriTrypDB) suggest that there are differences in the effect of RNAi against each of the three c subunit genes. RNAi against c-1 has the greatest effect on bloodstream forms with a smaller impact on procyclic cells. RNAi directed against c-2 shows a strong impact on both bloodstream and procyclic cells. RNAi directed against c-3 shows an effect on bloodstream but not procyclic cells. Further analysis of these cells may provide insight into the differences in function that the isoforms may have.

An additional interest might be in whether this N-terminal extension might result in the ability to inhibit the ATP synthase selectively. Subunit c is the site of interaction with not only DCCD, but more recently it has been determined that it is also central to the inhibition binding site for oligomycin [44]. Based on the crystal structure of oligomycin bound to the c_10_ ring from yeast ATP synthase, the authors postulate that the oligomycin binding site forms a framework for potential drug development based on the distinctions between mammalian and bacterial subunit c sequences. The *E. coli* sequence, for example, possesses only 2 of the conserved oligomycin interacting residues and is not sensitive to the inhibitor. The *T. brucei* sequence that corresponds to the conserved oligomycin domain contains a single amino acid difference with the consensus sequence from yeast to human (ILGFALTEA vs ILGFALSEA, but both we and others have shown that the *T. brucei* enzyme is less sensitive to oligomycin than yeast or mammals [15].

It has been suggested that only small changes in the subunit c backbone would be needed to alter inhibitor binding [44]. Given that the binding site of many inhibitors resides in this membrane component of the ATP synthase, further study of the F_0_ component of the ATP synthase, and subunit c in particular, is warranted.

## Supporting Information

Figure S1 **Antibodies raised to the three c subunit peptides do not show cross-reactivity.** Peptides selected from the region of dissimilarity (Figure 3) were spotted onto a nitrocellulose membrane followed by western blot analysis using the anti-peptide antibodies at 1∶2000, 1∶1000, and 1∶2500 for c-1, c-2, and c-3, respectively.
(TIF)

Table S1 **Primers were designed (IDT) to amplify c-1, c-2, and c-3 gene specific products.** Primers for Actin and Telomerase Reverse Transcriptase (TERT) were used as internal reference genes.
(DOCX)
