# Supplementary figures and images for: Three Distinct Isoforms of ATP Synthase Subunit c Are Expressed in T. brucei and Assembled into the Mitochondrial ATP Synthase Complex
Source: PLoS One. 2013 Jan 10;8(1):e54039. doi: 10.1371/journal.pone.0054039 (PMC3542316; doi:10.1371/journal.pone.0054039)

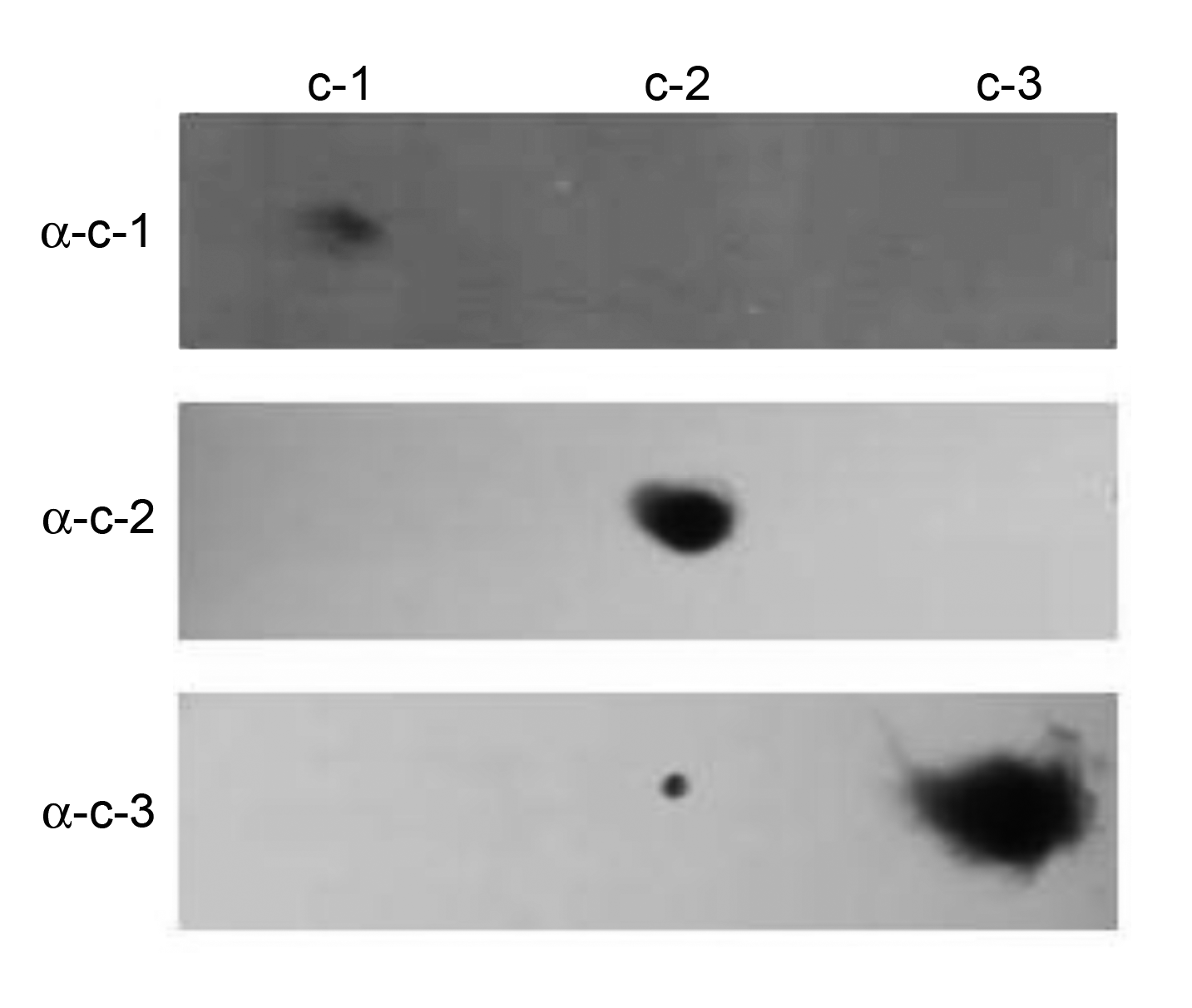

Supplement: Figure S1 — Antibodies raised to the three c subunit peptides do not show cross-reactivity. Peptides selected from the region of dissimilarity (Figure 3) were spotted onto a nitrocellulose membrane followed by western blot analysis using the anti-peptide antibodies at 1∶2000, 1∶1000, and 1∶2500 for c-1, c-2, and c-3, respectively. (TIF) [file pone.0054039.s001.tif]
