# Supplementary material for: Three Distinct Isoforms of ATP Synthase Subunit c Are Expressed in T. brucei and Assembled into the Mitochondrial ATP Synthase Complex
Source: PLoS One. 2013 Jan 10;8(1):e54039. doi: 10.1371/journal.pone.0054039 (PMC3542316; doi:10.1371/journal.pone.0054039)
Supplement: Table S1 — Primers were designed (IDT) to amplify c-1, c-2, and c-3 gene specific products. Primers for Actin and Telomerase Reverse Transcriptase (TERT) were used as internal reference genes. (DOCX) [file pone.0054039.s002.docx]

c-1 Forward- ^5’^ TTG CCC TTC AGT CCT CGC T ^3’^

c-1 Reverse- ^5’^ TGG CCA ATT GGG CTC GTA ACT T ^3’^

c-2 Forward- ^5’^ TCT CCA GTC TTC GAT TCG TCG T ^3’^

c-2 Reverse- ^5’^ TAA TGG CGC TGC ACA TAG GGT T ^3’^

c-3 Forward- ^5’^ CCT TGC CAT TCA GTC TTC TGT TCG ^3’^

c-3 Reverse- ^5’^ TAC ACA TCG GAC TGG CGA CCT TCA T ^3’^

Actin Forward- ^5’^ GTA CCA CTG GCA TTG TTC TCG ^3’^

Actin Reverse- ^5’^ CTT CAT GAG ATA TTC CGT CAG GTC ^3’^

TERT Forward-  ^5’^ GAG CGT GTG ACT TCC GAA GG ^3’^

TERT Reverse-  ^5’^ AGG AAC TGT CAC GGA GTT TGC ^3’^
